# Safety and efficacy of CT-guided percutaneous microwave ablation for stage I non-small cell lung cancer combined with moderate-to-severe pulmonary dysfunction

**DOI:** 10.3389/fonc.2026.1750853

**Published:** 2026-01-23

**Authors:** Yuxian Chen, Chengcheng Du, Chunhai Li, Hong Meng, Fanlei Kong

**Affiliations:** Department of Radiology, Qilu Hospital of Shandong University, Jinan, China

**Keywords:** complications, efficacy, microwave ablation, non-small cell lung cancer, pulmonary dysfunction, safety

## Abstract

**Introduction:**

To evaluate the safety and efficacy of microwave ablation (MWA) for stage I non-small cell lung cancer (NSCLC) in patients with pulmonary dysfunction.

**Methods:**

A retrospective study was conducted involving 1,635 patients who underwent MWA between January 2018 and December 2024. The observation group consisted of 42 patients with moderate-to-severe pulmonary dysfunction, defined as maximal voluntary ventilation (MVV) < 70% or forced expiratory volume in one second (FEV_1_) < 60%. The control group included 106 patients with normal lung function. The primary study endpoints were postoperative complications and length of hospitalization, while the secondary endpoint was progression-free survival (PFS).

**Results:**

The observation group had a median follow-up time of 25.5 months and a median survival period of 39 months, with 1-year and 3-year survival rates of 94.9% and 68.7%, respectively. The control group had a median follow-up time of 22 months and a median survival period of 44 months, with 1-year and 3-year survival rates of 98.1% and 72.3%, respectively. The median length of hospitalization was 6 days in both groups. The incidence of adverse reactions was 42.86% in the observation group and 33.96% in the control group, with no statistically significant difference between the two groups (p > 0.4099). In the observation group, statistically significant differences were identified with respect to smoking history, emphysema, and severe pulmonary dysfunction (p < 0.05). Univariate analysis indicated that patients with FEV_1_ (percentage of predicted value) < 55% and FEV_1_/VC < 70 were more prone to postoperative complications; however, multivariate analysis revealed no statistically significant differences.

**Discussion:**

MWA represents a safe, effective, and potential alternative therapeutic option for patients with stage I NSCLC and moderate-to-severe pulmonary dysfunction.

## 1 Introduction

Managing non-small cell lung cancer (NSCLC) patients with concurrent pulmonary dysfunction presents significant challenges, primarily stemming from poor prognosis and a relatively high incidence of severe postoperative complications (1). Anticancer therapy carries a high risk of fatal complications, which limits the available treatment options. While surgical resection is a potentially curative first-line treatment for early-stage NSCLC patients with comorbid pulmonary dysfunction, thoracic surgeons often face challenges in managing these patients—attributed to high postoperative morbidity and mortality, as well as poor prognosis. Additionally, comorbidities including limited cardiopulmonary reserve related to current or past tobacco use, high postoperative disability and mortality, poor patient prognosis, and evidence from studies demonstrating surgery’s adverse impact on lung function further complicate treatment (2). As a result, some patients are even denied surgical intervention due to poor lung function (3, 4).

CT-guided percutaneous microwave ablation (MWA) is an alternative treatment for patients with insufficient lung reserve who are not candidates for surgical resection (5, 6). MWA preserves lung parenchyma without causing permanent loss of lung function (7–9). Several studies have demonstrated that MWA is a novel minimally invasive technique that provides excellent local control of lung tumors and favorable prognostic outcomes for patient survival (10–13). Despite these advantages, the safety and efficacy of MWA in the treatment of pulmonary malignancies in patients with pulmonary dysfunction remain inadequately investigated. This study aimed to evaluate the safety and efficacy of MWA in patients with stage I NSCLC complicated by pulmonary dysfunction.

## 2 Materials and methods

This retrospective single-center study was approved by our institutional review board, which waived the requirement for informed consent. All patients provided written informed consent after being fully apprised of the potential risks and benefits of the procedure. Clinical data of the enrolled patients were retrieved from the hospital information system. Data collection and grouping were performed by independent physicians, and the researchers analyzing the data were blinded to the grouping assignments.

### 2.1 Pre−ablation evaluation

All patients underwent routine evaluation by interventional radiologists prior to MWA, including medical history review, physical examination, laboratory tests, and relevant imaging studies. Clinical staging was performed using thoracoabdominal contrast-enhanced CT or positron emission tomography/computed tomography (PET/CT). Anticoagulant and antiplatelet medications were temporarily discontinued for 1 to 7 days, depending on the type of medication. All patients also underwent routine complete blood count, coagulation function tests, biochemical tests, and an electrocardiogram before MWA.

### 2.2 Pulmonary function

A comprehensive pulmonary function assessment was performed for all patients in accordance with the guidelines established by the American Thoracic Society (ATS) and the European Respiratory Society (ERS) (14). Pulmonary function indicators—including forced vital capacity (FVC), forced expiratory volume in one second (FEV_1_), and maximum voluntary ventilation (MVV)—were measured for all patients. To account for differences in gender, age, height, and weight, both percent predicted values and raw data were used for comparison. ATS/ERS guidelines (2005/2019 editions) categorize ventilatory impairment into five grades: Mild (70% ≤ FEV_1_%pred ≤ 80%), Moderate (60% ≤ FEV_1_%pred ≤ 69%), Moderate-to-severe (50% ≤ FEV_1_%pred ≤ 59%), Severe (35% ≤ FEV_1_%pred ≤ 49%), and Very severe (FEV_1_%pred < 35%) (15). The study was conducted without supplementary oxygen.

### 2.3 Patients

Clinical data of 2,150 NSCLC patients treated with MWA between January 2018 and December 2024 were retrospectively analyzed. The inclusion criteria were as follows: (1) preoperative pulmonary function tests were performed; (2) histopathologically confirmed stage I NSCLC; (3) ablation margin > 5 mm; (4) lesions confined to the lung (unilateral or bilateral); and (5) age ≥ 18 years with an Eastern Cooperative Oncology Group (ECOG) performance status (PS) score ≤ 2. Exclusion criteria were: (1) regional lymph node metastasis or distant metastasis confirmed by contrast-enhanced CT, PET-CT, or contrast-enhanced magnetic resonance imaging; (2) receipt of other treatments (e.g., radioactive particle implantation, radiotherapy) either during the same admission or at different admissions; and (3) missing follow-up data. Among routine pulmonary function parameters, MVV is the most reliable indicator of pulmonary reserve function. It is generally accepted that surgery is safe when MVV exceeds 70% of the predicted value. Additionally, both FEV_1_ and diffusing capacity for carbon monoxide (DLCO) are recognized as independent prognostic factors (16). Typically, surgical resection is not recommended for patients with a preoperative FEV_1_% predicted < 50%, or a predicted postoperative FEV_1_ or carbon monoxide transfer factor < 40% (17, 18). Therefore, we concluded that mild pulmonary dysfunction had no significant impact on patient prognosis. In this study, the observation group included 42 patients with moderate-to-severe pulmonary dysfunction (defined by MVV < 70% or FEV_1_ ≤ 59%), while the control group included 106 patients with normal lung function who met the study criteria. This retrospective study was approved by our institutional review board (KYLL-202408-026), which waived the requirement for informed consent. Additionally, all patients provided written informed consent prior to MWA.

### 2.4 MWA procedure

MWA was performed using the ECO-100C device, which complies with the registration standard YZB/Country 3388–2011. The microwave transmission frequency was set at 2450 ± 50 MHz, with an adjustable output power range of 0–150 W. The microwave antenna had an effective length of 130–180 mm and an outer diameter of 15G–18G, and was equipped with a water-circulating cooling system to maintain a low surface temperature. CT imaging was utilized both to guide the MWA procedure and to assess its outcomes.

**Figure 1:**
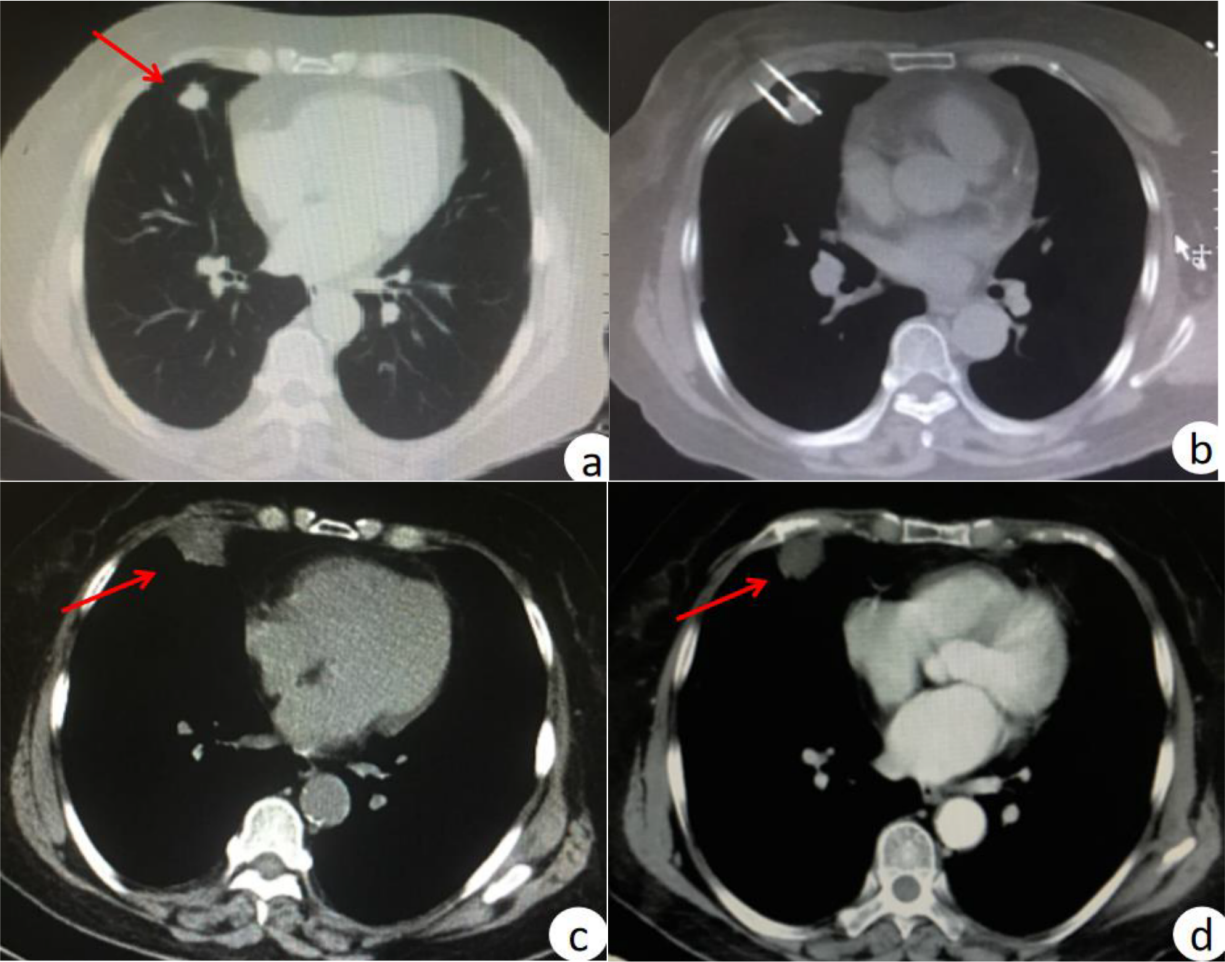
CT-guided MWA treatment for stage I non-small cell lung cancer. **(a)** before MWA treatment; **(b)** CT scan during MWA; **(c)** after MWA, the halo shadow was significantly larger than the lesion; **(d)** enhanced CT scan at 3 months post-MWA treatment showed no active lesions.

Before the procedure, the patient’s position (supine, lateral, prone, etc.) was determined via CT image analysis, and body surface marking lines were placed. The exact level and location of the lesion were identified using CT scans, and a planned needle insertion path was established. The entire procedure was performed under aseptic conditions, with local anesthesia administered at the puncture site. Under CT guidance, the ablation needle was carefully advanced into the lesion. A cable was then connected to link the ablation needle with the microwave generator and cooling circulation tube. Ablation duration and power levels were tailored to factors including lesion size, observed changes in the lesion, and patient tolerance. After MWA, an additional CT scan was performed to confirm that the ablation zone extended beyond the lesion area by a 5–10 mm margin. Following the patient’s safe transfer back to the ward, routine monitoring of heart rate, blood pressure, and oxygen saturation was initiated. A chest CT scan was scheduled 24–48 hours post-MWA for further evaluation (an example is shown in **Figure 1**).

### 2.5 Post-ablation management and follow-up

Patients were hospitalized for MWA and subsequent observation. Immediately after ablation, a chest CT was performed to assess the completeness of ablation and detect potential complications (e.g., pneumothorax, hemorrhage, pleural effusion). Technical success was defined as complete tumor coverage by the ablation zone with an adequate margin, confirmed by the immediate post-procedure CT scan. If ablation was successful and no urgent complications occurred, patients were transferred to the ward for observation. During this period, postoperative infections and wound pain were monitored, along with routine checks of heart rate, blood pressure, and oxygen saturation. Another chest CT was conducted 24–48 hours post-MWA; patients were discharged 2–3 days later if no further treatment for complications was required. Follow-up chest CTs were scheduled at 1, 3, 6, and 12 months post-procedure, with subsequent check-ups every 6 months.

### 2.6 Endpoint

The primary endpoints were postoperative complications and length of hospital stay. Complications were graded using the standardized Society of Interventional Radiology (SIR) system (19). Patients who died within 30 days post-procedure were categorized under SIR classification F. Major complications (corresponding to SIR classifications C–E) were defined as events causing significant morbidity and disability that required blood transfusions or interventional drainage; all other complications were classified as minor. The secondary endpoint was progression-free survival (PFS), defined as the time from the date of MWA treatment to the first occurrence of local tumor progression or distant metastasis, whichever came first.

### 2.7 Statistical analysis

Categorical data were compared using the χ² test, and continuous data were compared using the t-test. Kaplan-Meier survival curves were utilized for univariate analysis. A p-value < 0.05 was considered statistically significant. All statistical analyses were performed using SPSS software (version 27; SPSS, Inc., Chicago, IL, USA).

## 3 Results

### 3.1 Patients and tumor characteristics

**Figure 2:**
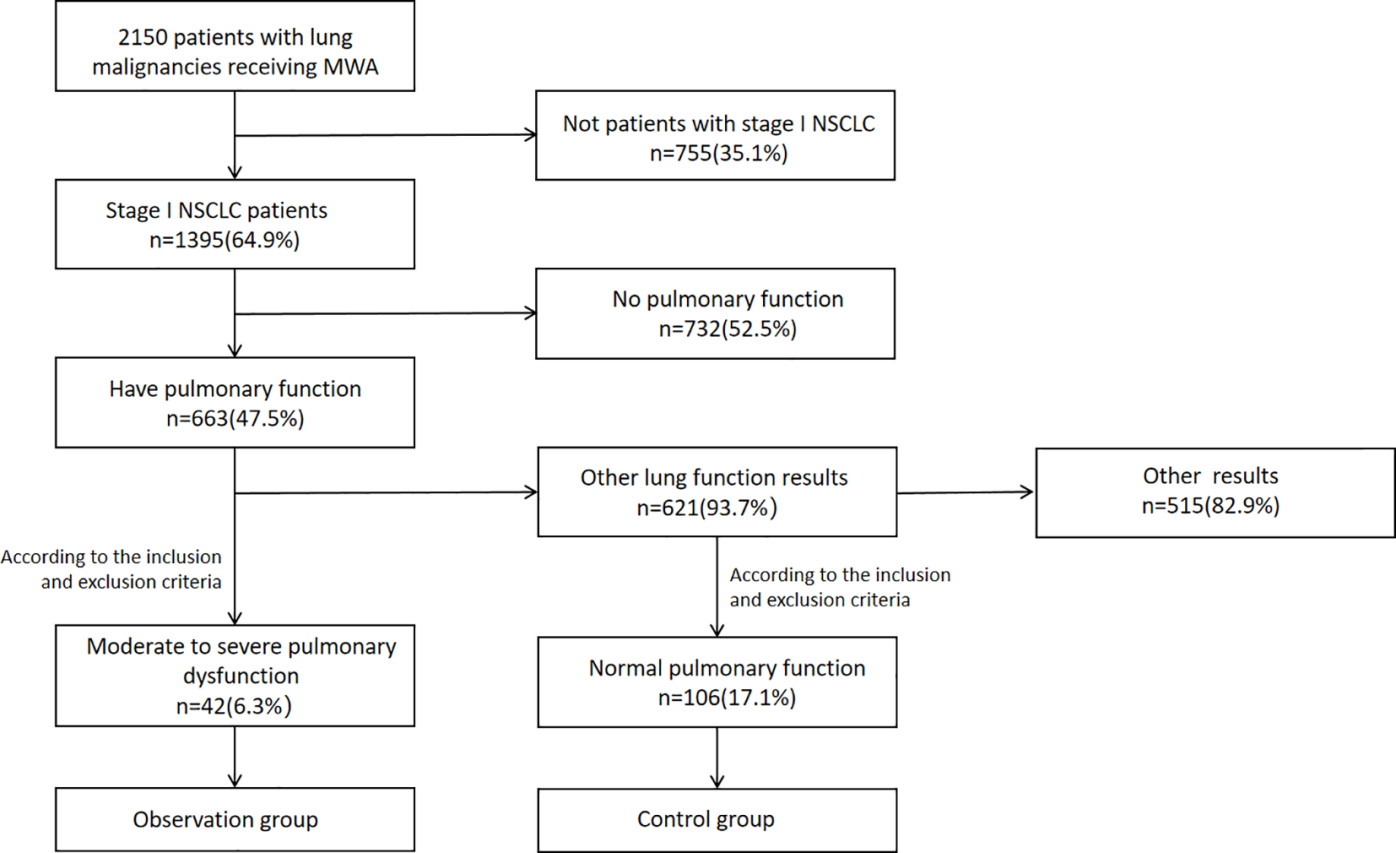
Flow diagram. MWA, microwave ablation; NSCLC, non-small cell lung cancer.

Among 663 stage I NSCLC patients who underwent MWA and had available lung function data, 148 were ultimately included in the analysis. The observation group and control group comprised 42 patients (42/148, 28.38%) and 106 patients (106/148, 71.62%), respectively (**Figure 2**). All patients had pathologically confirmed diagnoses; patient and tumor characteristics are detailed in **Table 1**. The 42 patients in the observation group collectively underwent 42 MWA procedures, treating a cumulative total of 48 lesions. Two cases required the simultaneous use of two ablation needles due to large lesion volumes, while six cases involved the simultaneous ablation of two lesions during a single treatment session. The control group comprised 106 patients who collectively underwent 106 MWA procedures, cumulatively treating 114 lesions. Among these, four cases required the simultaneous use of two ablation needles due to larger lesion volumes, while eight cases underwent the simultaneous ablation of two lesions during a single treatment session.

**Table 1:** Clinical characteristics of patients in two groups.

Characteristic	Observation group(n=42)	Control group(n=106)	*p*-value
Sex,n(%)			0.183
male	26(61.90)	51(48.11)	
female	16(38.10)	55(51.89)	
Age (y), median (range)	66.64(50-86)	63.18(49-86)	0.0783
Smoking history,n(%)			0.1528
yes	19(45.24)	33(31.13)	
no	23(54.76)	73(68.87)	
Location, n(%)			0.4940
upper left	11(26.19)	34(32.08)	
lower left	9(21.43)	14(13.21)	
upper right	15(35.71)	32(30.19)	
middle right	1(2.38)	8(7.55)	
lower right	6(14.29)	18(16.98)	
Tumor size (mm),mean ± SD	16.30 ± 6.40	14.55 ± 6.95	0.1587
Pathology,n(%)			0.2431
Adenocarcinoma	27(64.29)	80(75.47)	
Squamous cell carcinoma	15(35.71)	26(24.53)	

Thirty-one patients (31/42, 73.81%) presented with ≥ 1 comorbidity. The principal comorbidities included hypertension (19/42, 45.24%), coronary artery disease (15/42, 35.71%), emphysema (22/42, 52.38%), chronic obstructive pulmonary disease (16/42, 38.10%), and diabetes mellitus (3/42, 7.14%). Analysis of the association between smoking and various complications indicated that smoking exhibited a statistically significant correlation with emphysema and chronic obstructive pulmonary disease (p < 0.05). Preoperatively, the predicted FEV_1_ values ranged from 23.09% to 78.63% of predicted, with a mean of 56.43 ± 13.49% of predicted. MVV ranged from 31.32% to 69.82% of predicted, with a mean of 53.31 ± 12.53% of predicted (**Table 2**).

**Table 2:** Preoperative pulmonary function tests in the observation group, mean ± SD (range).

Indicators	Number of observations	Values
VC(% predicted)	42	78.42 ± 15.18(51.10-115.88)
FVC(% predicted)	42	80.08 ± 15.64(51.80-121.77)
FEV1(% predicted)	42	56.43 ± 13.49(23.09-78.63)
FEV1/FVC	42	73.14 ± 16.33(43.56-106.50)
FEV1/VC	42	71.68 ± 16.74(43.04-109.08)
MVV(% predicted)	42	54.14 ± 12.59(31.32-73.91)

VC, vital capacity; FVC, forced vital capacity; FEV_1_, forced expiratory volume in one second; MVV: maximum voluntary ventilation.

### 3.2 Adverse events and hospital length of stay

The incidence of adverse events in the observation group (42.86%, 18/42) was slightly higher than that in the control group (33.96%, 36/106), with no statistically significant difference (p = 0.4099). No procedure-related deaths occurred within 30 days post-MWA in either group. The grading of adverse events in the two groups is presented in **Table 3**. The mean length of hospital stay was 5.86 days in the observation group and 5.89 days in the control group, with no significant difference between the two groups (p = 0.9757).

**Table 3:** Adverse events of two groups.

Complication	Grade	Observation group (n=42)	Control group (n=106)	*p*-value
Major				
Pneumothorax	C	7/42(16.67%)	7/106(6.60%)	0.0692
Hemorrhage	C	0	1/106(0.94%)	> 0.999
Mortality	F	0	0	> 0.999
Minor				
Pneumothorax	A	7/42(16.67%)	17/106(16.04%)	> 0.999
Hemorrhage	A	2/42(4.76%)	10/106(9.43%)	0.5101
	B	1/42(2.38%)	3/106(2.83%)	> 0.999
Fever	A	2/42(4.76%)	2/106(1.89%)	0.3189
Chest pain	A	3/42(7.14%)	6/106(5.66%)	0.7136
Hospital length of stay,d,mean ± SD(range)		5.86 ± 1.73(3-11)	5.89 ± 1.56(3-10)	0.9757

Cox regression analysis incorporated vital capacity (VC, % predicted), FVC(% predicted), FEV_1_(% predicted), FEV_1_/FVC ratio, FEV_1_/VC ratio, and MVV (% predicted). Univariate analysis indicated that patients with FEV_1_ (percentage of predicted value) < 55% and FEV_1_/VC < 70 were more prone to postoperative complications; however, multivariate analysis revealed no statistically significant differences (**Table 4**).

**Table 4:** Cox regression analysis of prognostic factors for adverse events.

Variables	Univariate analysis		Multivariate analysis	
	HR (95% CI)	*p*-value	HR (95% CI)	*p*-value
VC(% predicted)	0.887(0.311-2.533)	0.823	1.023(0.247-4.230)	0.975
FVC(% predicted)	0.890(0.201-3.935)	0.877	0.412(0.058-2.926)	0.375
FEV1(% predicted)	4.069(1.389-11.922)	0.011	4.184(0.888-19.716)	0.070
FEV1/FVC	1.983(0.767-5.125)	0.158	0.571(0.140-2.326)	0.434
FEV1/VC	3.742(1.073-13.044)	0.038	3.038(0.645-14.300)	0.160
MVV(% predicted)	2.619(0.327-20.942)	0.364	2.126(0.257-17.600)	0.484

### 3.3 Survival rate

The median follow-up duration was 25.5 months (range: 10–53 months) for the observation group and 22 months (range: 10–65 months) for the control group, with no statistically significant difference between the two groups (p = 0.1478). No patients were lost to follow-up.

**Figure 3:**
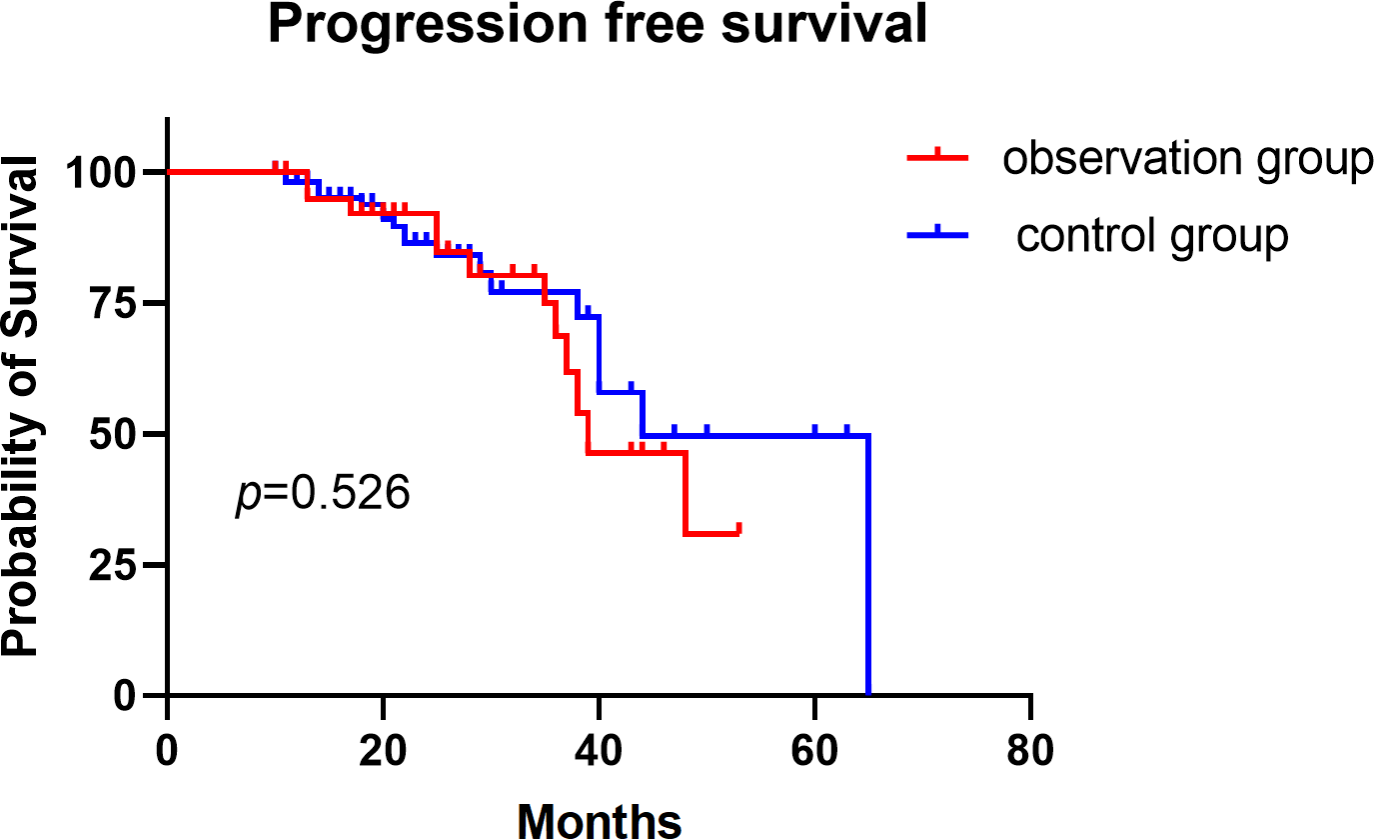
Progression-free survival for stage I non-small cell lung cancer in two groups.

In the observation group, the median progression-free survival (PFS) was 39 months, with 1-year and 3-year PFS rates of 94.9% and 68.7%, respectively. In the control group, the median PFS was 44 months, with 1-year and 3-year PFS rates of 98.1% and 72.3%, respectively. However, the difference in PFS between the two groups was not statistically significant (p = 0.526; **Figure 3**).

Cox regression analysis included variables such as gender, age, smoking history, pathology, tumor size, vital capacity (VC, % predicted), FVC(% predicted), FEV_1_(% predicted), FEV_1_/FVC ratio, FEV_1_/VC ratio, and MVV (% predicted). Both univariate and multivariate analyses revealed no statistically significant associations between these variables and PFS (p > 0.05; **Table 5**).

**Table 5:** Cox regression analysis of prognostic factors for PFS.

Variables	Univariate analysis		Multivariate analysis	
	OR (95% CI)	*p*-value	OR (95% CI)	*p*-value
Gender	1.025(0.323-3.254)	0.966	0.775(0.122-4.909)	0.787
Age	0.809(0.238-2.748)	0.734	0.601(0.083-4.360)	0.614
Smoke history	0.646(0.203-2.054)	0.459	0.189(0.027-1.321)	0.093
Pathology	2.782(0.608-12.729)	0.187	4.126(0.669-25.432)	0.127
Tumor size(mm)	1.495(0.423-5.276)	0.532	1.537(0.195-12.092)	0.683
VC(% predicted)	1.626(0.493-5.365)	0.425	1.276(0.171-9.531)	0.812
FVC(% predicted)	2.166(0.569-8.236)	0.257	1.907(0.206-17.625)	0.569
FEV1(% predicted)	2.403(0.716-8.067)	0.156	1.263(0.533-5.312)	0.107
FEV1/FVC	1.248(0.4.-3.889)	0.702	0.350(0.011-11.296)	0.554
FEV1/VC	0.773(0.248-2.413)	0.658	0.321(0.028-3.624)	0.358
MVV(% predicted)	2.507(0.010-6.536)	0.414	1.976(0.847-11.125)	0.988

## 4 Discussion

This retrospective study evaluated the safety and efficacy of MWA in the treatment of stage I NSCLC in patients with moderate-to-severe pulmonary dysfunction, with patients with normal lung function serving as the control group. The results demonstrated that MWA was well-tolerated and efficacious in patients with pulmonary dysfunction, with no statistically significant difference in safety outcomes between the two groups.

While the impact of impaired pulmonary reserve function on lung cancer surgery remains challenging to quantify, it is now widely accepted that surgical resection is not recommended for patients with MVV < 70% of predicted, preoperative FEV_1_ < 50% of predicted, or predicted postoperative FEV_1_ or carbon monoxide transfer factor < 40% of predicted. Several studies (3, 20) have confirmed that a significant proportion of patients with anatomically resectable NSCLC—including some early-stage cases who would otherwise be eligible for surgical resection but are deemed unsuitable for surgery due to poor lung function—typically receive alternative treatment regimens such as chemotherapy and/or radiotherapy (21, 22). However, these modalities often fail to achieve curative outcomes and may be associated with systemic toxicities that further compromise the functional status of patients with preexisting pulmonary impairment.

For patients unable or unwilling to undergo surgery due to elevated risks, MWA emerges as a promising alternative. As prior research indicates, thermal ablation (including MWA) may be safe and effective for high-risk populations, such as those with a history of single pneumonectomy (23–25). However, the safety and efficacy of MWA specifically for stage I NSCLC in patients with severe pulmonary dysfunction have not been fully elucidated. Compared with surgical resection, MWA’s minimally invasive nature—characterized by smaller incisions, reduced parenchymal trauma, and preservation of lung tissue—renders it particularly suitable for patients with pulmonary dysfunction and other comorbidities. Recent studies have demonstrated no significant deterioration in lung function following thermal ablation for stage IA NSCLC (7, 23, 24), which aligns with our finding that no pulmonary function indicators were statistically associated with adverse outcomes. This suggests that MWA avoids the iatrogenic lung function decline inherent to surgical resection, a key advantage for patients with limited respiratory reserve.

In terms of safety, the incidence of adverse events in the observation group (42.86%) was slightly higher than that in the control group (33.96%), but the difference was not statistically significant (p = 0.4099). Pneumothorax was the most common adverse event, occurring in 33.33% of the observation group and 22.64% of the control group (p = 0.179), with rates consistent with those reported in previous MWA studies for lung malignancies (26–28). Notably, no procedure-related deaths occurred within 30 days postoperatively in either group, with few major complications (SIR grades C–E). This confirms that MWA maintains favorable safety profiles even in patients with moderate-to-severe pulmonary impairment. Its minimally invasive nature minimizes disruption to the compromised respiratory system and reduces the risk of life-threatening complications such as severe pneumothorax or major hemorrhage. Furthermore, the median hospital stay was 6 days in both groups, with mean durations of 5.86 and 5.89 days, respectively. This outcome further underscores the favorable tolerability of MWA, supporting its viability as a feasible option for patients with limited functional reserve.

In this study, univariate analysis indicated that FEV_1_ < 55% (predicted) and FEV_1_/VC < 70 were significantly associated with an increased risk of postoperative complications. However, this association was not retained in multivariate analysis, potentially due to multicollinearity among pulmonary function indices and insufficient statistical power from the relatively small sample size. Despite the lack of statistical significance in multivariate analysis, the clinical relevance of these parameters should not be discounted: FEV_1_ directly reflects airway patency and expiratory capacity, while the FEV_1_/VC ratio is a robust indicator of airway obstruction severity (29, 30) Both are physiologically linked to postoperative respiratory complications, as impaired expiratory function can exacerbate atelectasis, increase the risk of infection, and prolong recovery. Thus, combined assessment of FEV_1_ and FEV_1_/VC remains a practical tool for identifying high-risk patients, enabling targeted interventions such as preoperative respiratory rehabilitation, enhanced intraoperative monitoring (e.g., real-time oxygenation and ventilation assessment), and proactive postoperative management of air leaks.

A statistically significant association was observed between smoking history and emphysema, chronic obstructive pulmonary disease (COPD), and severe pulmonary dysfunction (p < 0.05). Smoking triggers inflammatory and oxidative stress responses in the airways and alveoli; these responses induce damage to the lung parenchyma—including alveolar wall destruction and reduced lung elastic fibers—which ultimately leads to emphysema. In turn, these pathological changes serve as the foundation for the development of severe pulmonary dysfunction (31, 32). This finding also underscores the importance of smoking cessation counseling in clinical practice. Emphysema is widely recognized as a primary driver of COPD and progressive pulmonary dysfunction. However, in the present cohort study, no statistically significant association was detected between emphysema and severe pulmonary dysfunction (p = 0.332). This discrepancy may stem from heterogeneity in emphysema severity, confounding comorbidities, or variations in treatment histories—all of which could obscure the inherent association—or from underlying differences in pathogenesis, pathophysiological processes, and clinical manifestations between the two conditions. Furthermore, limitations in sample size may have compromised the statistical power to detect such an association. Thus, we cannot simply conclude that emphysema necessarily progresses to severe pulmonary dysfunction, nor can we overlook the potential role of emphysema as a risk factor for severe pulmonary dysfunction in clinical decision-making.

In this study, the median progression-free survival (PFS) of the observation and control groups was 39 and 44 months, respectively, with 1-year PFS rates of 94.9% vs. 98.1% and 3-year PFS rates of 68.7% vs. 72.3%—values consistent with prior studies of MWA for inoperable stage I NSCLC. For example, a National Cancer Database analysis reported 1-, 3-, and 5-year relative survival rates of 90.0%, 58%, and 37% for thermal ablation (33), while Peng et al. observed a 1-year PFS of 93.7% in elderly (>70 years) inoperable stage I NSCLC patients (34), and Ni et al. reported a median PFS of 36 months in a similar cohort (35). The lack of a statistically significant difference in PFS between our two groups (p = 0.526) further confirms that pulmonary dysfunction does not compromise the efficacy of MWA.

This study is limited by its retrospective, single-center design and small sample size. Additionally, the absence of a surgical control group may compromise the generalizability and statistical reliability of the findings. Future research should involve large-scale, multicenter studies incorporating surgical control groups to clarify the relative advantages of MWA. Furthermore, the predictive value of pulmonary function indicators for postoperative complications warrants further validation through optimized variable selection and the inclusion of indicator interaction terms.

In summary, this study demonstrates that CT-guided percutaneous MWA represents a safe and effective treatment option for stage I NSCLC patients with moderate to severe pulmonary impairment. This therapy achieves favorable local tumor control with an acceptable complication rate, and survival outcomes remain unaffected compared to patients with normal pulmonary function.

## Data Availability

The raw data supporting the conclusions of this article will be made available by the authors, without undue reservation.
